# Patterns of Antibiotic Use in Hospitalized COVID-19 Patients and Association with Secondary Infections and Outcomes

**DOI:** 10.3390/antibiotics15030240

**Published:** 2026-02-25

**Authors:** Diana-Maria Mateescu, Ana-Olivia Toma, Dragos-Mihai Gavrilescu, Adrian-Cosmin Ilie, Eduard Florescu, Ovidiu Rosca, Cristian Oancea, Stela Iurciuc, Camelia-Oana Muresan, Alexandra Enache

**Affiliations:** 1 Doctoral School, Department of General Medicine, “Victor Babes” University of Medicine and Pharmacy, Eftimie Murgu Square 2, 300041 Timisoara, Romania; diana.mateescu@umft.ro; 2 Discipline of Dermatology, “Victor Babes” University of Medicine and Pharmacy, Eftimie Murgu Square 2, 300041 Timisoara, Romania; toma.olivia@umft.ro; 3 Center for the Morphologic Study of the Skin (MORPHODERM), “Victor Babes” University of Medicine and Pharmacy, Eftimie Murgu Square 2, 300041 Timișoara, Romania; 4 Department of Orthodontics, Dental District, Zăgazului 3, One Floreasca Vista, 014261 Bucharest, Romania; dr.gavrilescu@outlook.com; 5 Department of Public Health and Sanitary Management, “Victor Babes” University of Medicine and Pharmacy, Eftimie Murgu Square 2, 300041 Timisoara, Romania; ilie.adrian@umft.ro; 6 Centre for Translational Research and Systems Medicine, Faculty of Medicine, “Victor Babes” University of Medicine and Pharmacy, Eftimie Murgu Square 2, 300041 Timișoara, Romania; 7 Pulmonology Department, “Victor Babes” University of Medicine and Pharmacy, Eftimie Murgu Square 2, 300041 Timisoara, Romania; 8 Methodological and Infectious Diseases Research Center, Department of Infectious Diseases, “Victor Babes” University of Medicine and Pharmacy, Eftimie Murgu Square 2, 300041 Timisoara, Romania; ovidiu.rosca@umft.ro; 9 Department of Infectious Diseases, “Victor Babes” University of Medicine and Pharmacy, Eftimie Murgu Square 2, 300041 Timisoara, Romania; 10 Center for Research and Innovation in Personalised Medicine of Respiratory Diseases (CRIPMRD), Pulmonology University Clinic, “Victor Babes” University of Medicine and Pharmacy, Eftimie Murgu Square 2, 300041 Timisoara, Romania; 11 Cardiology Department, “Victor Babes” University of Medicine and Pharmacy, Eftimie Murgu Square 2, 300041 Timisoara, Romania; 12 Legal Medicine, Timisoara Institute of Legal Medicine, Eftimie Murgu Square 2, 300041 Timisoara, Romania; enache.alexandra@umft.ro; 13 Ethics and Human Identification Research Center, “Victor Babes” University of Medicine and Pharmacy, Eftimie Murgu Square 2, 300041 Timisoara, Romania; 14 Discipline of Forensic Medicine, Bioethics, Deontology, and Medical Law, Department of Neuroscience, “Victor Babes” University of Medicine and Pharmacy, Eftimie Murgu Square 2, 300041 Timisoara, Romania

**Keywords:** antibiotic exposure, COVID-19 hospitalization, secondary infections, antimicrobial stewardship, multidrug-resistant pathogens, AWaRe classification, hospital-acquired infection, clinical outcomes, intensive care, antimicrobial resistance

## Abstract

**Background/Objectives**: Antibiotic exposure is highly prevalent in patients hospitalized with COVID-19, yet the relationship between specific prescribing patterns, microbiologically confirmed secondary infections, and clinical outcomes remains incompletely understood, particularly in settings with high antimicrobial resistance. **Methods**: This single-center retrospective observational cohort included 395 consecutive adults hospitalized with RT-PCR-confirmed COVID-19 in a tertiary infectious diseases hospital. Data on demographics, comorbidities, baseline disease severity, antimicrobial prescribing (timing, WHO AWaRe class, duration, monotherapy/combination, escalation/de-escalation), microbiological results, and outcomes were extracted from electronic records and the microbiology information system. The primary outcome was microbiologically confirmed secondary infection; secondary outcomes were ICU admission, invasive mechanical ventilation, length of stay, and in-hospital mortality. Multivariable logistic regression and survival analyses assessed associations between antibiotic exposure and outcomes. **Results**: Overall, 88.4% of patients received systemic antibiotics, predominantly initiated within 24 h of admission and mostly empirical; 58.7% received combination regimens, with frequent use of Watch/Reserve agents. Secondary infections occurred in 28.4% of patients, were hospital-acquired in 82.1%, and involved multidrug-resistant organisms in 41.1% of cases. Any antibiotic exposure was independently associated with secondary infection (adjusted odds ratio, aOR 2.15; 95% CI 1.42–3.27), while prolonged therapy (≥7 days), Watch/Reserve use, and early initiation showed additional risk gradients. Antibiotic exposure was also associated with higher odds of ICU admission, invasive mechanical ventilation, prolonged hospitalization, and in-hospital mortality after adjustment. **Conclusions**: In this real-world COVID-19 cohort, broad and largely empirical antibiotic use was common and strongly associated with hospital-acquired, often multidrug-resistant secondary infections and worse clinical outcomes. These findings highlight the need for reinforced antimicrobial stewardship focusing on restrictive early broad-spectrum use, AWaRe-guided agent selection, systematic 48–72 h reassessment with de-escalation, and minimization of treatment duration.

## 1. Introduction

Antibiotic exposure may modify outcomes in hospitalized patients with COVID-19 through multiple mechanisms. Broad-spectrum agents are able to disrupt host microbiota, promote colonization with multidrug-resistant organisms (MDROs), and heighten susceptibility to hospital-acquired infections, including ventilator-associated pneumonia, bloodstream infections, and *Clostridioides difficile* infection [1,2,3]. Severe SARS-CoV-2 infection is also frequently associated with prolonged hospitalization, immune dysregulation, lymphopenia, and the widespread use of systemic corticosteroids, all of which further increase the risk of secondary infections [4,5]. Despite extensive global attention to COVID-19 management, comprehensive analyses linking specific antibiotic prescribing patterns to microbiologically confirmed secondary infections and clinical outcomes remain limited and methodologically heterogeneous across health service environments [6,7,8].

A detailed understanding of the determinants of antibiotic initiation, escalation, and duration has become essential for strengthening antimicrobial stewardship during severe viral illness, where diagnostic uncertainty and clinical pressure frequently favor broad-spectrum empirical therapy [7,9]. The pandemic highlighted differences in stewardship practices, including variation in conformity to guidelines and the influence of local epidemiology on prescribing behavior. Secondary infections have been consistently associated with higher morbidity, mortality, ICU admission, and healthcare resource use [2,4,8], yet the specific contribution of antecedent antibiotic exposure to these outcomes in COVID-19 remains insufficiently defined, with large observational cohorts and meta-analyses reporting heterogeneous and sometimes conflicting associations between antibiotic use, secondary infection, and mortality [1,2,6,10,11,12,13].

Existing studies report inconsistent associations between antibiotic use and clinical outcomes, partly due to differences in hospital practices, variability in microbiological testing, and incomplete adjustment for underlying disease severity [2,6,8,9,10,11,12,13,14]. Large multicenter cohorts and systematic reviews, including the ISARIC WHO CCP-UK study and several meta-analyses, have mainly described overall antibiotic prescribing prevalence and crude associations with mortality, without integrating detailed WHO AWaRe-based categorization or longitudinal exposure metrics. Other investigations evaluating bacterial co-infection and secondary infection in hospitalized COVID-19 patients often used antibiotic exposure as a binary variable, did not consistently apply microbiologically confirmed endpoints as primary outcomes, and only partially adjusted for baseline severity and competing risks [2,6,8,9,10,11,12,13,14]. Only a limited number of investigations have systematically evaluated comprehensive antibiotic prescribing patterns—considering timing, class selection, treatment duration, and combination therapy—in parallel with microbiological findings and primary clinical outcomes [1,6,9]. In-depth evaluation of these factors is needed to guide antimicrobial stewardship tactics and improve therapeutic decision-making during future viral respiratory epidemics.

This study examines antibiotic use among hospitalized COVID-19 patients, focusing on prescribing patterns and their associations with microbiologically documented secondary infections, disease severity, and clinical outcomes. Through integrating antimicrobial exposure data with laboratory parameters, microbiological cultures, and clinical indicators, the study intends to determine whether antibiotic use increases the risk of secondary infections and adverse outcomes, and to identify modifiable stewardship targets. These data are intended to support evidence-based antimicrobial decision-making and inform post-pandemic strategies aiming to mitigate antimicrobial resistance in acute-care settings. In resource-constrained environments, where microbiological capacity may be restricted, characterizing prescribing patterns may also afford essential context and enhance the applicability of stewardship recommendations throughout diverse medical systems [7,14]. In this analysis, antibiotic exposure was operationalized using the WHO AWaRe framework by assigning each systemic agent to its AWaRe category and characterizing both the highest AWaRe category reached during the hospitalization and the cumulative days of therapy within the Access, Watch, and Reserve groups [15,16,17]. This approach distinguishes between initial empirical choices, subsequent escalation into higher-risk categories, and the overall AWaRe-weighted antibiotic burden at the patient level.

To our knowledge, this is one of just a few studies integrating detailed AWaRe-based antibiotic categorization, treatment duration, timing of initiation, and escalation/de-escalation with the primary outcome of microbiologically confirmed secondary infection and predefined secondary clinical outcomes in a tertiary infectious diseases hospital with a documented high antimicrobial resistance burden, characterized by elevated rates of multidrug-resistant Gram-negative bacilli and a high proportion of WHO Watch and Reserve agents in institutional surveillance and national AMR/AMU reports [16,17,18,19,20,21].

## 2. Results

### 2.1. Patient Characteristics

All 395 included patients underwent at least partial microbiological evaluation during hospitalization (blood cultures, respiratory samples, urine cultures, and/or *Clostridioides difficile* testing), in line with routine practice in our tertiary infectious diseases center for moderate-to-severe COVID-19. Direct comparison of baseline characteristics between included patients and those excluded for lacking any microbiological data was not feasible, as detailed screening logs were not retrievable from the retrospective electronic records. To explore potential selection bias toward more severe cases (who are more likely to undergo extensive diagnostic workup), we examined proxy indicators within the included cohort. Patients with documented positive blood cultures (*n* = 27) had similar demographics to those without (mean age 70.1 ± 11.4 vs. 70.4 ± 11.1 years; male sex 55.6% vs. 53.8%), but higher admission CRP levels and a greater need for supplemental oxygen, consistent with more severe illness and greater diagnostic intensity in sicker patients. These patterns support that the cohort reflects typical hospitalized moderate-to-severe COVID-19 cases in our setting; multivariable models therefore adjusted for baseline severity markers (oxygen requirement, inflammatory biomarkers) to mitigate residual bias.

A total of 395 adult patients hospitalized with RT-PCR-confirmed COVID-19 were included in the final analysis after application of eligibility criteria. The mean age was 70.3 ± 11.2 years, with 58.2% (*n* = 230) male and 41.8% (*n* = 165) female patients, and 61.0% (*n* = 241) resided in urban areas. Mean body mass index (BMI) was 29.4 ± 5.6 kg/m^2^, and 42.5% (*n* = 168) met criteria for obesity (BMI ≥ 30 kg/m^2^) (Table 1).

COVID-19 vaccination coverage was as follows: 48.1% (*n* = 190) were fully vaccinated (≥2 doses), predominantly with BNT162b2 (Pfizer; 72.1% of vaccinated), 12.4% (*n* = 49) were partially vaccinated (single dose, mostly Ad26.COV2.S [Johnson & Johnson]; 55.1%), and 39.5% (*n* = 156) were unvaccinated. A smoking history was documented in 24.3% (*n* = 96), while 9.1% (*n* = 36) were immunosuppressed due to underlying conditions or chronic immunosuppressive therapy (Table 1).

Comorbidity burden was high. Cardiovascular disease was present in 62.3% (*n* = 246), including arterial hypertension in 58.7% (*n* = 232; mean admission blood pressure 138.4 ± 18.2/72.5 ± 12.1 mmHg) and ischemic heart disease in 28.1% (*n* = 111). Diabetes mellitus was present in 32.7% (*n* = 129), predominantly type 2 (89.9% [*n* = 116]), followed by type 1 (7.8% [*n* = 10]) and newly diagnosed diabetes at admission (2.3% [*n* = 3]). Chronic kidney disease was recorded in 18.0% (*n* = 71), chronic obstructive pulmonary disease in 14.2% (*n* = 56), and obesity as above (Table 1).

Baseline COVID-19 severity was categorized as moderate-to-severe in 68.4% (*n* = 270) according to NIH/WHO criteria. Mean admission SpO_2_ was 90.2 ± 3.8%, and 92.7% (*n* = 366) required oxygen supplementation at presentation. Baseline inflammatory and coagulation markers were frequently abnormal: leukocyte count 9.8 ± 4.7 × 10^3^/µL, C-reactive protein (CRP) 98.5 ± 72.3 mg/L, procalcitonin 0.52 ± 0.81 ng/mL, ferritin 812.4 ± 592.6 ng/mL, D-dimer 1.42 ± 1.18 µg/mL, and interleukin-6 (IL-6) 42.3 ± 38.7 pg/mL; 84.6% (*n* = 334) exhibited at least one elevated inflammatory marker, as shown in Table 1.

At admission, most patients presented with moderate-to-severe COVID-19 according to NIH/WHO criteria, and a substantial proportion required supplemental oxygen. Baseline severity is described in Table 1 through oxygen requirement, SpO_2_, and inflammatory and coagulation markers (CRP, D-dimer, IL-6, ferritin), rather than composite NEWS2 or SOFA scores, which could not be reliably calculated for the entire cohort due to missing essential components, as detailed in the Methods section. At admission, mean CRP and procalcitonin levels were higher among patients who ultimately received antibiotics compared with those managed without antibiotics, consistent with greater baseline inflammatory burden in the antibiotic-exposed group (Supplementary Materials, Table S2)

### 2.2. Antibiotic Prescribing Patterns

Overall, 88.4% (*n* = 349/395) of patients received at least one systemic antibiotic during hospitalization (Table 2). Among antibiotic-exposed patients, therapy was initiated early (≤24 h from admission) in 72.5% (*n* = 253/349) and later (>24 h) in 27.5% (*n* = 96/349). Mean antibiotic treatment duration was 8.2 ± 3.1 days (median 7 days, IQR 5–10), defined as patient-level days of therapy (DOT), i.e., the number of calendar days during which a patient received at least one systemic antibiotic, irrespective of the number of concomitant agents. Monotherapy was used in 41.3% (*n* = 144/349), whereas 58.7% (*n* = 205/349) received combination regimens. Treatment modifications occurred frequently, with escalation in 32.1% (*n* = 112/349) and de-escalation in 21.5% (*n* = 75/349) (Table 2). Escalation and de-escalation were defined a priori based on spectrum changes and microbiological clarification, as detailed in the Methods Section (Section 4.4.1).

**Figure 1:**
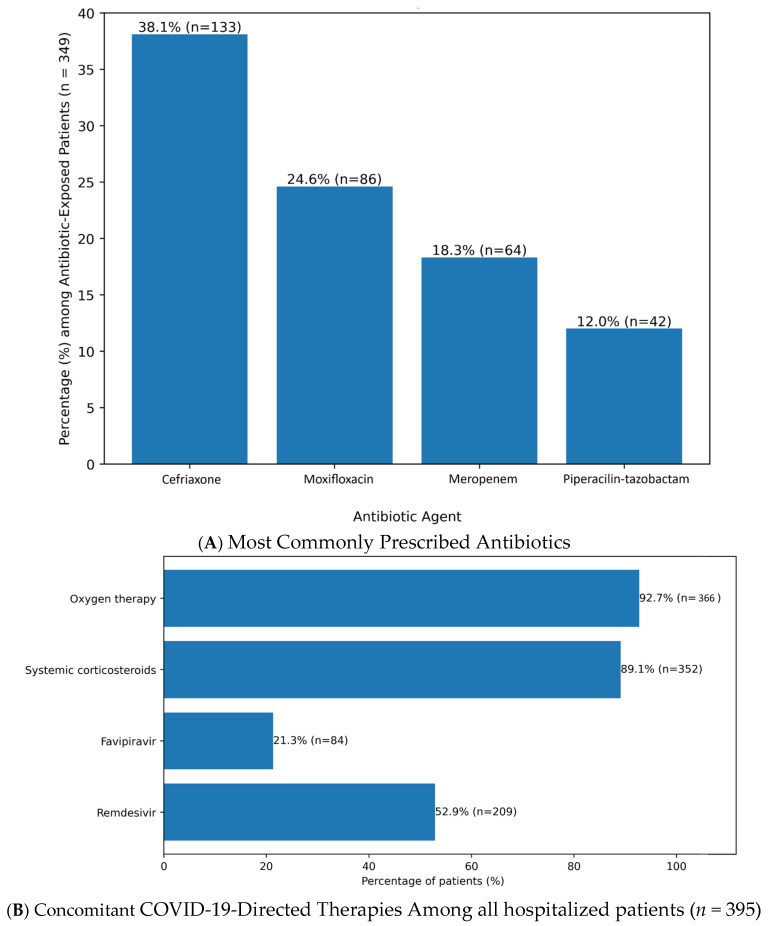
(**A**) Most frequently prescribed systemic antibiotics among antibiotic-exposed hospitalized COVID-19 patients (*n* = 349), highlighting predominant use of broad-spectrum agents within the WHO AWaRe Watch and Reserve categories. Values are expressed as number and percentage of patients. (**B**) COVID-19-directed therapies administered during hospitalization, including antiviral agents, systemic corticosteroids, and oxygen supplementation, in the overall study cohort (*n* = 395). Data are presented as number and percentage of patients.

According to the WHO AWaRe classification, Access antibiotics were prescribed in 28.4% (*n* = 99/349), Watch antibiotics in 54.7% (*n* = 191/349), and Reserve antibiotics in 16.9% (*n* = 59/349). The most commonly used agents were ceftriaxone (38.1% [*n* = 133/349]), moxifloxacin (24.6% [*n* = 86/349]), meropenem (18.3% [*n* = 64/349]), and piperacillin–tazobactam (12.0% [*n* = 42/349]), as in Figure 1A.

Empirical prescribing predominated: 81.4% (*n* = 284/349) of antibiotic courses were initiated without microbiological confirmation available at the time of the prescribing decision (including both fully empirical starts and empirical therapy with cultures pending), while 18.6% (*n* = 65/349) met criteria for culture-guided (definitive) therapy, i.e., initiation or major modification after pathogen identification and/or susceptibility results became available (Table 2).

Concomitant COVID-19-directed therapies included remdesivir in 52.9% (*n* = 209/395; mean duration 4.8 ± 1.2 days), favipiravir in 21.3% (*n* = 84/395; mean 6.1 ± 2.4 days), systemic corticosteroids in 89.1% (*n* = 352/395; mean 7.4 ± 3.0 days), and oxygen therapy in 92.7% (*n* = 366/395; mean 6.5 ± 4.2 days), as shown in Figure 1B.

### 2.3. Secondary Infections and Microbiological Findings

**Figure 2:**
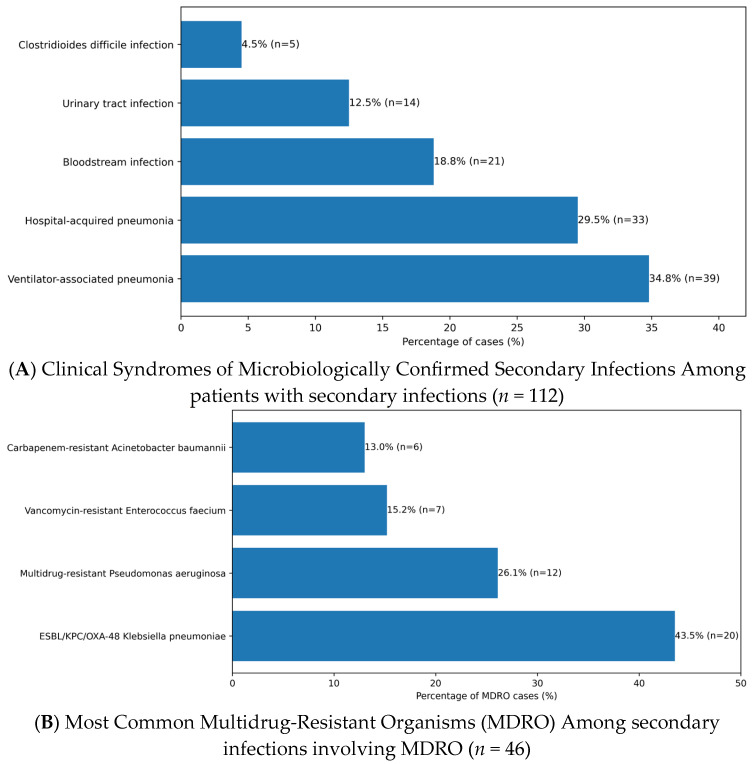
(**A**) Distribution of microbiologically confirmed secondary infection syndromes among hospitalized COVID-19 patients who developed superinfection (*n* = 112). Data are expressed as number and percentage of cases. (**B**) Distribution of multidrug-resistant organisms (MDROs) isolated from microbiologically confirmed secondary infections among hospitalized COVID-19 patients (*n* = 46). Data are presented as number and percentage of MDRO cases.

Microbiologically confirmed secondary infections occurred in 28.4% (*n* = 112/395) of patients. Among these, 82.1% (*n* = 92/112) met criteria for hospital-acquired infection (onset >48 h after admission). The most frequent syndromes were ventilator-associated pneumonia (34.8% [*n* = 39/112]), hospital-acquired pneumonia (29.5% [*n* = 33/112]), bloodstream infection (18.8% [*n* = 21/112]), urinary tract infection (12.5% [*n* = 14/112]), and *Clostridioides difficile* infection (4.5% [*n* = 5/112]), as shown in Table 3 and Figure 2A.

Co-infections at admission were less frequent, documented in 7.6% (*n* = 30/395), predominantly bacterial pneumonia (63.3% [*n* = 19/30]), and were analyzed separately from secondary infections. Multidrug-resistant organisms (MDROs) were isolated in 41.1% (*n* = 46/112) of patients with secondary infections. The most common MDROs were ESBL/KPC/OXA-48–producing *Klebsiella pneumoniae* (43.5% [*n* = 20/46]), multidrug-resistant *Pseudomonas aeruginosa* (26.1% [*n* = 12/46]), vancomycin-resistant *Enterococcus faecium* (15.2% [*n* = 7/46]), and carbapenem-resistant *Acinetobacter baumannii* (13.0% [*n* = 6/46]) (Table 3, Figure 2B).

Secondary infections were significantly more frequent among antibiotic-exposed patients compared with those not receiving antibiotics (31.2% [*n* = 109/349] vs. 6.5% [*n* = 3/46]; *p* < 0.001, χ^2^ test). However, the exact onset date of secondary infections (clinical or microbiological) was not systematically recorded in the source documents, precluding a precise quantification of the time from antibiotic initiation to secondary infection onset. Consequently, the observed relationship between antibiotic exposure and secondary infections reflects an association during the index hospitalization, and no causal direction can be inferred from these data.

Microbiological sampling practices reflected underlying disease severity. Patients requiring ICU admission or invasive mechanical ventilation more frequently underwent repeated blood and respiratory cultures than ward patients, in keeping with routine diagnostic escalation in critical illness. Because of this severity-linked sampling pattern, we conducted a sensitivity analysis restricted to patients with at least one documented blood culture; the adjusted association between antibiotic exposure and secondary infection was consistent with the main findings, supporting the robustness of the observed relationship despite potential detection bias. In a supplementary descriptive analysis, the proportion of patients with microbiologically confirmed bloodstream infection was similar across ICU and antibiotic-exposure strata (Supplementary Materials, Table S1). Specifically, bloodstream infection occurred in 0 of 21 ICU patients (0.0%) versus 27 of 374 non-ICU patients (7.2%), and in 20 of 285 antibiotic-exposed patients (7.0%) versus 7 of 110 non-exposed patients (6.4%). These patterns suggest that overt differences in bloodstream infection rates across key clinical strata were limited, although they do not directly quantify microbiological sampling intensity.

### 2.4. Clinical Outcomes

The primary endpoint (microbiologically confirmed secondary infection) occurred in 28.4% (*n* = 112/395) as detailed above. Among secondary outcomes, 22.0% (*n* = 87/395) required ICU admission, with a mean ICU length of stay of 8.9 ± 5.3 days. Invasive mechanical ventilation was required in 15.7% (*n* = 62/395), with a mean duration of 7.2 ± 4.1 days, as in Table 4.

The overall in-hospital length of stay was 10.4 ± 6.8 days (median 9 days; IQR 5–14). In-hospital mortality was 12.7% (*n* = 50/395). Pulmonary complications, including pleural effusion and thromboembolic events, occurred in 38.2% (*n* = 151/395). Sepsis or septic shock was documented in 14.9% (*n* = 59/395). Additional complications included urinary tract infection not meeting criteria for secondary infection (9.1% [*n* = 36/395]), enterocolitis (5.3% [*n* = 21/395]), and in-hospital cardiac arrest (4.1% [*n* = 16/395]), as shown in Table 4.

### 2.5. Associations Between Antibiotic Use and Outcomes

In multivariable logistic regression adjusted for age, comorbidities, baseline COVID-19 severity, systemic corticosteroid use, and duration of hospitalization, any antibiotic exposure was independently associated with an increased risk of secondary infection (aOR 2.15; 95% CI 1.42–3.27; *p* < 0.001; Table 5).

**Figure 3:**
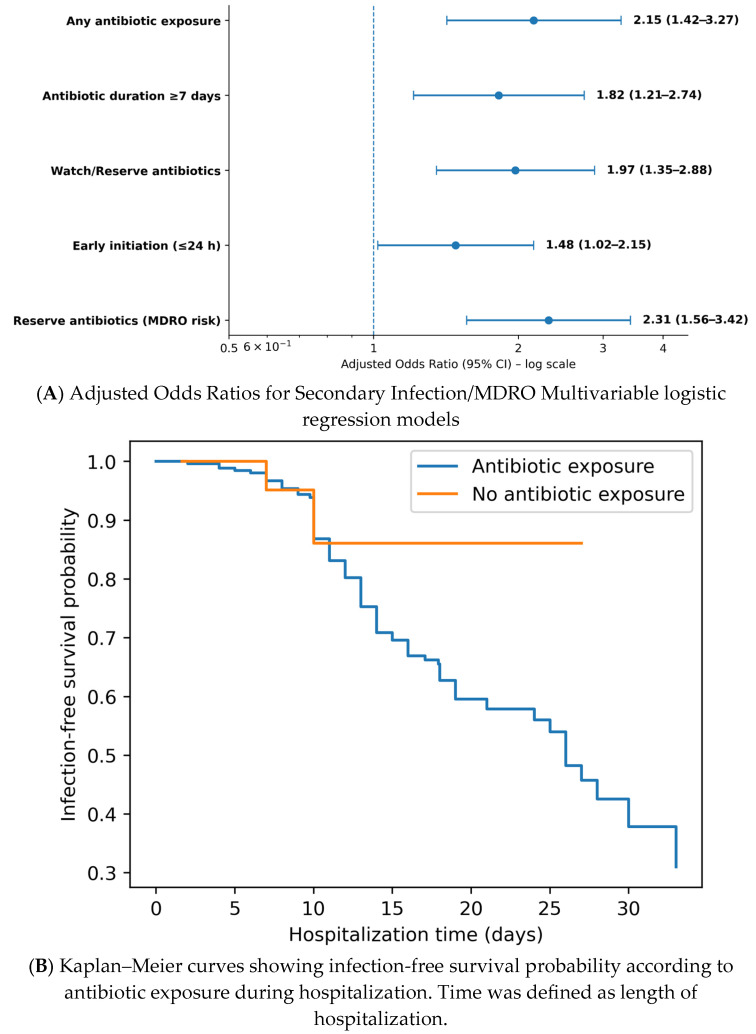
(**A**) Multivariable-adjusted association between exposure to Reserve antibiotics and the risk of multidrug-resistant organism (MDRO) colonization or infection among hospitalized COVID-19 patients. Results are expressed as adjusted odds ratios (aORs) with 95% confidence intervals. (**B**) Kaplan–Meier curves for time to an infection-related event (sepsis and/or a documented bloodstream isolate) according to antibiotic exposure during hospitalization in the study cohort. Time was defined as length of hospitalization because the exact onset date of secondary infections was not consistently available. Differences between groups were assessed using the log-rank test (*p* = 0.94).

Specific prescribing patterns showed distinct risk gradients. Antibiotic duration >7 days was associated with higher odds of secondary infection (aOR 1.82; 95% CI 1.21–2.74; *p* = 0.004), as was the use of Watch/Reserve antibiotics (aOR 1.97; 95% CI 1.35–2.88; *p* < 0.001) and early initiation (≤24 h) (aOR 1.48; 95% CI 1.02–2.15; *p* = 0.039). Exposure to Reserve antibiotics was strongly associated with subsequent MDRO colonization or infection (defined as isolation of an MDRO either from a site meeting criteria for secondary infection or from surveillance and diagnostic cultures obtained during hospitalization) (aOR 2.31, 95% CI 1.56–3.42; *p* < 0.001), as shown in Figure 3A.

Beyond secondary infection, antibiotic exposure remained associated with adverse clinical outcomes after adjustment, including ICU admission (aOR 1.76; 95% CI 1.18–2.62; *p* = 0.005), invasive mechanical ventilation (aOR 1.93; 95% CI 1.27–2.94; *p* = 0.002), prolonged hospital stay >10 days (aOR 1.64; 95% CI 1.12–2.41; *p* = 0.011), and in-hospital mortality (aOR 1.89; 95% CI 1.22–2.93; *p* = 0.004), as in Table 5.

Kaplan–Meier analysis using hospitalization duration as the time scale showed similar event-free survival between antibiotic-exposed and non-exposed patients (log-rank *p* = 0.94), as shown in Figure 3B. Because the exact onset date of secondary infections was not consistently recorded, time-to-event analyses were anchored to length of stay and the event definition was restricted to infection-related endpoints captured in the dataset (sepsis and/or a documented bloodstream isolate).

**Figure 4:**
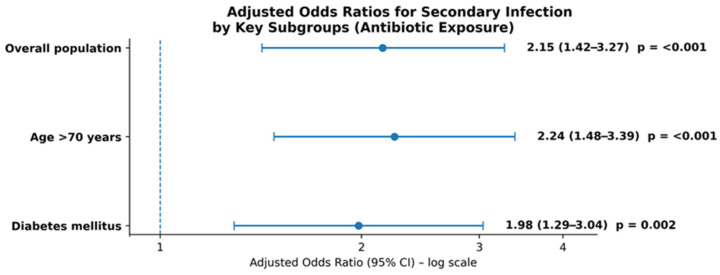
Forest plot of multivariable-adjusted odds ratios for microbiologically confirmed secondary infection associated with antibiotic exposure in the overall population and predefined high-risk subgroups (age >70 years and diabetes mellitus). Results are presented as adjusted odds ratios (aORs) with 95% confidence intervals.

Subgroup analyses suggested increased vulnerability among elderly patients (>70 years) and those with diabetes mellitus. In patients aged >70 years, antibiotic exposure was associated with higher odds of secondary infection (aOR 2.24; 95% CI 1.48–3.39; *p* < 0.001), while in patients with diabetes mellitus the association was similar (aOR 1.98; 95% CI 1.29–3.04; *p* = 0.002), as in Figure 4. No statistically significant interaction was observed according to vaccination status (*p* = 0.12, χ^2^ test).

## 3. Discussion

In this retrospective real-world cohort of 395 consecutively hospitalized adults with laboratory-confirmed COVID-19, antibiotic exposure was almost universal (88.4%), with most prescriptions initiated early (≤24 h) and predominantly empirical. Secondary infections were microbiologically documented in 28.4% of patients and were largely hospital-acquired (onset >48 h), with a substantial MDRO burden (41.1% of secondary infections). Significantly, antibiotic exposure remained independently associated with higher odds of secondary infection and multiple adverse clinical outcomes after adjustment for demographic factors, comorbidity burden, COVID-19 severity, systemic corticosteroid use, and hospitalization duration. Collectively, these data support a clinically meaningful risk gradient linking broad and prolonged antibiotic therapy—particularly Watch/Reserve agents—to microbiologically confirmed superinfection, MDRO emergence, and worsened in-hospital prognosis. This interpretation is consistent with high-quality evidence syntheses documenting persistent antibiotic overuse in COVID-19 despite low bacterial co-infection prevalence at admission and with stewardship-focused recommendations advocating restrictive empiric strategies and early reassessment [10,11,15,22,23].

### 3.1. Antibiotic Use in Hospitalized COVID-19: Persistent Overexposure

A central finding of this study is the magnitude of antibiotic prescribing: 88.4% of patients received systemic antibiotics, whereas only 7.6% had documented co-infection at admission, and 28.4% developed microbiologically confirmed secondary infections during hospitalization. This pronounced prescribing–infection mismatch mirrors international systematic reviews and living meta-analyses, which show that confirmed bacterial co-infection at presentation is uncommon. At the same time, antibiotic exposure in hospitalized COVID-19 patients remains disproportionately high across several healthcare settings [10,11].

In this cohort, empirical prescribing predominated (81.4% of courses), frequently as combination regimens and with substantial reliance on broad-spectrum therapy. Similar prescribing patterns were described in extensive multicenter studies in which early empiric antibacterial therapy was common despite very low prevalence of confirmed community-onset bacterial co-infection, supporting the conclusion that routine broad-spectrum therapy at admission is rarely justified absent robust clinical, radiologic, or biomarker evidence. Evidence-based recommendations further recommend short courses with early clinical and microbiologic reassessment when bacterial infection is not supported [22,23].

From a stewardship perspective, the heavy use of broad-spectrum agents is especially worrying in light of the WHO AWaRe classification, which stratifies antibiotics according to resistance-selection potential and explicitly recommends restricting Watch and Reserve agents to clearly defined indications and emphasizing Access antibiotics where appropriate [15].

### 3.2. Secondary Infections: Burden, Timing, and MDRO Profile

Secondary infections occurred in 28.4% of patients, and 82.1% of events were hospital-acquired (onset >48 h), strengthening the concept that bacterial and fungal superinfections in COVID-19 predominantly represent complications of prolonged hospitalization and advanced supportive care rather than frequent drivers at admission. This time-based distribution is consistent with ICU cohorts reporting substantial healthcare-associated infection incidence in critically ill COVID-19 patients, particularly among those requiring invasive ventilation and prolonged organ support [24,25].

The infection spectrum observed here was typical of severe viral pneumonia settings: ventilator-associated/hospital-acquired pneumonia accounted for most events, followed by bloodstream and urinary tract infections and *Clostridioides difficile* infection. Notably, 41.1% of secondary infections involved MDROs, in line with critical care evidence reporting frequent MDR etiologies in COVID-19-associated HAIs and stressing the compounded burden of antimicrobial exposure, device utilization, and pandemic-era strain on infection prevention and control [18,24,25].

### 3.3. Antibiotic Exposure as an Independent Predictor of Secondary Infection and MDRO

A significant strength of this analysis is the linkage of granular antibiotic exposure patterns (timing, duration, AWaRe category) to microbiologically confirmed endpoints rather than clinical suspicion alone. After multivariable adjustment, antibiotic exposure remained independently associated with secondary infection, with coherent exposure–response gradients indicating higher risk with prolonged therapy, Watch/Reserve agents, and early initiation. These outcomes are consistent with systematic evidence demonstrating that antibiotic use in COVID-19 frequently exceeds the prevalence of bacterial infection and may contribute to downstream nosocomial infections and AMR selection, particularly in high-acuity care settings [10,11,19].

Mechanistically, broad-spectrum antibiotics disrupt commensal microbiota and weaken colonization resistance, facilitating overgrowth and translocation of opportunistic and resistant organisms, chiefly within the gut—an established reservoir for MDRO colonization. In parallel, pandemic-era analyses of antibiotic utilization support the plausibility that excessive antimicrobial exposure increases selective pressure and susceptibility to MDRO-related complications. COVID-19 microbiome studies additionally demonstrate that antibiotic-associated dysbiosis is linked to microbial translocation and bacteremia, while broader gut microbiome alterations correlate with disease severity and immune dysfunction. Together, these data support the plausibility that antibiotic exposure may represent a potentially modifiable marker of susceptibility to infectious complications, although our observational design does not allow us to distinguish definitively between antibiotic-related effects and the influence of baseline severity or care intensity [26,27,28].

### 3.4. Antibiotics and Adverse Clinical Outcomes

Beyond secondary infections, antibiotic exposure remained associated with ICU admission, need for invasive mechanical ventilation, prolonged hospitalization, and in-hospital mortality after multivariable adjustment. This pattern is likely multifactorial, reflecting confounding by indication, mediation through secondary infection, and potential direct harm pathways related to microbiome disruption, antibiotic-associated adverse events, and AMR ecology [19,26].

Time-to-event analyses based on hospitalization duration did not show statistically significant crude differences between exposure groups. Nevertheless, multivariable models identified antibiotic exposure patterns as independent predictors of secondary infection and adverse outcomes, in a manner compatible with a ‘risk-amplification’ framework. However, given the retrospective observational design, these associations are likely influenced by confounding by indication, underlying clinical severity, and treatment intensity, and should not be interpreted as proof of a direct causal effect of antibiotics on downstream outcomes. This interpretation is consistent with post-pandemic stewardship analyses noting that COVID-19-related antibiotic overuse may have accelerated AMR emergence and supports the requirement for coordinated stewardship and surveillance approaches [19,26,27,28,29,30].

### 3.5. Consequences for Antimicrobial Stewardship and Clinical Pathways

The present findings translate into pragmatic antimicrobial stewardship priorities for future waves of COVID-19 and other viral respiratory epidemics. These priorities are primarily grounded in existing stewardship evidence and guidelines, with our cohort providing supportive associative data from a high-resistance setting rather than establishing causality. First, early empirical broad-spectrum therapy should be restricted to patients with a high pre-test probability of bacterial infection (e.g., focal consolidation, purulent sputum, septic shock, or biomarker profiles strongly suggestive of bacterial sepsis), rather than used as a default in hospitalized patients with viral pneumonia. Evidence-based antibacterial guidance and extensive multicenter cohort data support this restrictive approach and emphasize early reassessment when diagnostic data become available [22,23].

Second, AWaRe-guided prescribing should be operationalized to give priority to Access agents where appropriate and to reserve Watch/Reserve antibiotics for clearly justified scenarios presenting documented or strongly suspected resistant pathogens. Third, hospitals should implement mandatory 48–72 h antibiotic “time-outs,” integrating microbiological results, radiology, clinical trajectory, and inflammatory markers, facilitating systematic de-escalation or discontinuation when bacterial infection is not supported [15,29].

Fourth, antibiotic durations should be minimized whenever clinically feasible, as the exposure–risk gradients observed with prolonged therapy correspond to the wider post-pandemic stewardship imperative to reduce antibiotic-associated harm and resistance selection. In addition, sepsis-oriented best practices emphasize repeated reassessment to de-escalate and avoid unnecessary prolonged courses, therewith strengthening the role of structured antimicrobial reviews in high-risk hospitalized populations [29,31].

At the population level, surveillance data indicate that achieving and maintaining a high level of antibiotic access continues a measurable and actionable stewardship target, and antibiotic consumption monitoring can be used to benchmark and evaluate hospital- and system-level stewardship interventions [16,17,32].

Given the high MDRO proportion among secondary infections, stewardship interventions must be tightly integrated with infection prevention and control strategies (hand hygiene adherence, ventilator/catheter bundles, environmental cleaning, and appropriate isolation precautions) to limit cross-transmission in high-stress settings. Addressing prescribing, diagnostics, infection control, and surveillance in parallel is essential to lessening the compounded burden of nosocomial infection and AMR [16,17,18,24,25,29,30,32].

### 3.6. High-Risk Subgroups and Clinical Stratification

Subgroup analyses indicated a stronger association between antibiotic exposure and secondary infection among older adults (>70 years) and patients with diabetes mellitus. This is biologically plausible given immunometabolic dysregulation, chronic inflammation, endothelial injury, and heightened vulnerability to severe COVID-19 and nosocomial complications. Extensive population-based studies have demonstrated markedly increased COVID-19 mortality among individuals with both type 1 and type 2 diabetes [33,34].

Instead of defaulting to longer and broader antibiotic courses in these vulnerable groups, patients may benefit more from intensified microbiological evaluation, closer clinical monitoring, and structured stewardship review to avoid iatrogenic amplification of infection and AMR risk. Tailored stewardship algorithms incorporating age, comorbidity burden, baseline severity, and local AMR ecology may help balance the competing risks of undertreating true bacterial infections and overtreating with broad-spectrum agents [15,22,23,29].

### 3.7. Strengths and Limitations

This study shows several strengths that improve its credibility and relevance. The inclusion of a consecutive, unselected real-world cohort of hospitalized adults with RT-PCR-confirmed COVID-19 reduces selection bias and mirrors routine clinical practice, thereby increasing external validity in comparable tertiary care settings. The research delivers an in-depth analysis of antibiotic exposure, detailing timing, WHO AWaRe classification, treatment duration, monotherapy versus combination therapy, and escalation or de-escalation. This procedure permits a fine-grained analysis of prescribing patterns rather than a simple binary exposure comparison. Systematic linkage of these exposure metrics with confirmed secondary infections, including pathogen and resistance profiles, delivers a strong framework to clarify the relationships among prescribing practices, multidrug-resistant organism (MDRO) epidemiology, and clinical outcomes [10,11,15,19,22,23,24].

From a methodological perspective, the study improves internal validity through employing multivariable logistic regression and survival models adjusted for key confounders, including age, comorbidities, baseline COVID-19 severity, corticosteroid use, and length of stay. Modeling antibiotic duration as a time-dependent covariate addresses immortal time and time-related biases, which frequently complicate studies of treatment exposure and hospital-acquired infection. The direction of associations is consistent across key endpoints, such as secondary infection, ICU admission, invasive mechanical ventilation, length of hospitalization, and in-hospital mortality. The explicit use of AWaRe classification facilitates comparability with international stewardship benchmarks and supports the translation of findings into policy and guideline discussions [15,16,26,29,31].

Several limitations should be considered when interpreting these outcomes. The retrospective, single-center design restricts causal inference, and temporal associations between antibiotic exposure and secondary outcomes do not establish directionality. In particular, the lack of systematically recorded onset dates for secondary infections limits our ability to confirm that these infections occurred after antibiotic initiation, further reinforcing that our stewardship-related findings should be interpreted as associative rather than causal. The potential for unmeasured confounding persists. Conducted in a tertiary infectious diseases hospital with specific resistance patterns, microbiology capacity, and stewardship practices, the study’s setting may limit generalizability to institutions with different case mixes, resistance profiles, resources, or prescribing cultures. While definitions of healthcare- and device-associated infections were aligned with major surveillance frameworks, operational differences, local diagnostic thresholds, and sampling practices may influence event rates and MDRO distributions [18,24,25,35,36].

Residual and unmeasured confounding factors remain concerning despite multivariable adjustment. Certain aspects of disease severity and clinical decision-making, such as dynamic oxygen requirements, radiological changes, frailty, or the clinician’s assessment of bacterial superinfection, may not be entirely captured by recorded scores and laboratory markers. Microbiological sampling practices may vary by patient, with more severely ill individuals, those in the ICU, or those receiving mechanical ventilation undergoing more frequent and invasive cultures. This can increase the detection of secondary infections and MDRO colonization, potentially inflating associations between antibiotic exposure, ICU care, and secondary infection. In the most critically ill patients, treatment and diagnostic intensity often overlap. Reliance on routine data may introduce exposure and outcome misclassification, including timing errors, incomplete documentation of dose adjustments, or challenges when distinguishing colonization from infection in certain respiratory cultures. These factors may bias effect estimates in either direction [18,24,25,35,36].

The time-dependent dynamics of antibiotic exposure bring in extra methodological complexity. Antibiotic use evolves throughout hospitalization, with patients admitted for longer durations more likely to receive multiple antibiotic courses and develop hospital-acquired infections. Conversely, patients who deteriorate rapidly may die before these events occur. While time-to-event models that include antibiotic duration as a time-varying covariate reduce the risks of immortal time bias and time confounding, some degree of estimation distortion may persist. Competing risks, such as early mortality preceding secondary infection, are challenging to address with the available data and models fully. Furthermore, the study did not methodically assess diagnostic stewardship tools, such as standardized procalcitonin algorithms or multiplex respiratory panels, which may influence antibiotic initiation and discontinuation, thus affecting exposure patterns and outcomes [37,38,39].

Related to this, antibiotic duration, length of hospitalization, and clinical severity are tightly interrelated. Longer antibiotic courses frequently occur in patients with more severe disease and prolonged stays, and these factors independently increase the risk of hospital-acquired infection. In our primary logistic models, adjusting for duration of hospitalization was intended to account for heterogeneous time at risk, but we acknowledge that length of stay may also act as a mediator or collider on the causal pathway between antibiotic exposure, secondary infection, and adverse outcomes. Consequently, these adjusted estimates should be interpreted as describing associations rather than unbiased causal effects, and our findings are best viewed within a risk-amplification framework rather than as proof that antibiotics directly cause the observed outcomes.

Third, the primary outcome of microbiologically confirmed secondary infection is prone to detection bias. Microbiological sampling was clinician-driven and more intensive in patients with greater illness severity, ICU admission, or invasive mechanical ventilation, which increases the likelihood of detecting secondary infections and MDRO colonization in these groups and may inflate the observed association between antibiotic exposure, critical care, and secondary infection. To address this concern, we performed sensitivity analyses restricting the cohort to patients with documented microbiological sampling (operationalized as having at least one blood culture), and observed similar adjusted effect estimates for the association between antibiotic exposure and secondary infection, suggesting that our findings are robust to differential sampling intensity, although residual bias cannot be fully excluded. In addition, the proportion of patients with microbiologically confirmed bloodstream infection was broadly similar across ICU and antibiotic-exposure strata (Supplementary Materials, Table S1), indicating that large systematic differences in detected bloodstream infection rates between exposure groups are unlikely, even though we could not formally compare the exact number of cultures per patient. This practice may result in underestimating secondary infections among low-risk patients and overestimating them among those receiving intensive monitoring. Colonization pressure, prior MDRO carriage, and environmental elements were not systematically assessed, limiting the ability to distinguish antibiotic effects from background transmission. The study was conducted during the COVID-19 pandemic, a period defined by unique care pressures, staffing changes, and potential temporary alterations in stewardship practices. These factors may restrict the applicability of findings to post-pandemic conditions [18,20,21,24,25].

We acknowledge that we were unable to compute NEWS2 and SOFA scores for the entire cohort because several mandatory components of these scores were not systematically collected at admission. However, we mitigated this limitation by providing a detailed description of baseline severity using NIH/WHO severity categories, oxygen requirement, and comprehensive laboratory profiles, which are available for all patients. A key limitation is the potential for selection bias, as patients with more severe disease or clinical deterioration were more likely to undergo comprehensive microbiological testing, while milder cases without cultures may have been excluded. This could lead to overestimation of secondary infection rates and of the strength of their association with antibiotic exposure. Because data were retrospectively extracted from electronic records without preserved full screening logs, characteristics of excluded patients could not be directly compared. We mitigated this risk through multivariable adjustment for baseline severity surrogates, proxy analyses within the included cohort, and sensitivity testing, which supported the robustness of the findings; nevertheless, residual bias cannot be fully excluded, and prospective designs with systematic microbiological sampling in all hospitalized COVID-19 patients would be required to eliminate this concern. Fourth, we used complete-case analyses without multiple imputation, which may introduce bias if data were not missing completely at random, although key covariates had low missingness.

In spite of these limitations, the central message remains intact. Caution is justified to avoid overinterpreting causality as well as generalizability. Antimicrobial resistance is still common in European acute-care hospitals. The real-world findings of this study are consistent with existing evidence supporting shorter, targeted antibiotic courses for suspected bacterial pneumonia. This points out the stewardship objective: to avoid early broad-spectrum therapy lacking substantial evidence, to reexamine therapy at 48 to 72 h, and to minimize antibiotic duration once clinical stability is achieved [16,17,29,31,32,40].

## 4. Materials and Methods

### 4.1. Study Design and Setting

We conducted a retrospective observational cohort study including consecutive adult patients hospitalized with confirmed COVID-19 in a tertiary infectious diseases center between 1 March 2020 and 31 December 2024. This interval encompasses several major national pandemic waves, including the pre-vaccination wild-type/Alpha phase (2020–early 2021), the Delta-dominant period (mid-2021), and subsequent Omicron-predominant periods (2022–2024), which were associated with evolving vaccination coverage and treatment practices at the population level. Thus, the cohort captures real-world prescribing behavior across multiple pandemic contexts rather than a single wave. The hospital provides advanced respiratory and critical care services and maintains an integrated microbiology laboratory with continuous surveillance for antimicrobial resistance.

### 4.2. Eligibility Criteria

Patients were eligible for inclusion if they met all of the following criteria: (1) age ≥ 18 years; (2) laboratory-confirmed SARS-CoV-2 infection by real-time reverse transcription polymerase chain reaction (RT-PCR); (3) hospitalization for COVID-19-related illness for at least 24 h.

We excluded patients admitted for non-COVID-19 primary diagnoses, those transferred to or from external facilities without complete antimicrobial records, and individuals lacking microbiological data necessary for classification of secondary infections. Because complete screening logs for all admissions during the study period were not preserved in the electronic record system, the exact number of patients excluded due to lacking microbiological data could not be reliably reconstructed, and direct comparison of baseline characteristics between included and excluded patients was not feasible. Cohort assembly is depicted in Supplementary Figure S1, a STROBE-style flow diagram summarizing screening, application of eligibility criteria, and inclusion of the final analytic cohort (*n* = 395).

### 4.3. Data Sources and Variables Collected

Data were retrieved from electronic medical records and the hospital microbiology information system. For each patient, we collected: (i) demographic and baseline characteristics: age, sex, comorbidities, smoking status, and immunosuppression; (ii) markers of disease severity at admission: respiratory status, need for oxygen supplementation, vital signs (blood pressure, heart rate, pulse oximetry), and laboratory parameters (white blood cell count, CRP, procalcitonin, ferritin, D-dimer, IL-6, liver and kidney function tests); (iii) antimicrobial prescribing data: indication, timing of initiation (within 24 h of admission or later), agent(s), route of administration, duration of therapy, and whether escalation or de-escalation occurred; (iv) microbiological results: blood cultures, respiratory cultures (endotracheal aspirates, BAL), urine cultures, stool toxin testing for *Clostridioides difficile*, and susceptibility profiles of all isolates, classified using EUCAST breakpoints; (v) clinical outcomes: development of microbiologically confirmed secondary infection, ICU transfer, need for invasive mechanical ventilation, length of stay, and in-hospital mortality.

Clinical data collected at admission included demographics, comorbidities, vital signs (blood pressure, heart rate, pulse oximetry), oxygen requirement, and key laboratory parameters (white blood cell count, CRP, D-dimer, IL-6, ferritin, liver and kidney function tests). Although NEWS2 and SOFA scores are commonly used to summarize baseline severity in COVID-19 cohorts, several indispensable components (systematic respiratory rate, temperature, Glasgow Coma Scale, PaO_2_/FiO_2_ ratio, vasopressor use, bilirubin levels and urine output) were not consistently available for all admissions, precluding a robust and unbiased computation of these composite scores for the entire cohort. Therefore, baseline COVID-19 severity was operationalized using NIH/WHO COVID-19 severity categories together with oxygen requirement, SpO_2_ on admission, and admission biomarker profiles, which are fully available for the cohort. NEWS2 and SOFA scores were not systematically computed for the cohort for the reasons detailed in the Data Sources and Variables Collected subsection.

Inflammatory biomarkers (CRP, procalcitonin, ferritin, D-dimer, IL-6) were systematically recorded at admission; however, repeated measurements at the exact time of antibiotic initiation were not consistently available in the retrospective dataset and could not be robustly summarized as time-updated covariates.

Secondary infections were classified as hospital-acquired if identified >48 h after admission, consistent with CDC and ECDC surveillance criteria. Co-infections detected at presentation were recorded but analyzed separately.

### 4.4. Definitions

#### 4.4.1. Antibiotic Exposure

Antibiotic exposure was defined as the administration of any systemic antibacterial agent during hospitalization. Prescribing patterns were analyzed across the following dimensions: timing: early (≤24 h from admission) vs. late (>24 h); antibiotic class: categorized according to WHO AWaRe classification (Access, Watch, Reserve); treatment duration: continuous days of therapy (DOT); regimen structure: monotherapy vs. combination therapy; modifications: escalation (addition of broader-spectrum agents) or de-escalation (narrowing spectrum or discontinuation after microbiological clarification). DOT was calculated at the patient level as the number of calendar days during which a patient received at least one systemic antibiotic; for patients receiving combination therapy, each calendar day with any systemic antibiotic exposure counted as one DOT, irrespective of the number of concomitant agents.

For the purposes of this analysis, antibiotic prescribing was further categorized according to the microbiological context at initiation as follows: (i) fully empirical therapy, when antibiotics were started before any microbiological specimens were obtained or when no cultures were collected at all; (ii) empirical therapy with cultures pending, when appropriate cultures were obtained before or on the same calendar day as antibiotic initiation, but microbiological and susceptibility results were not yet available at the time of the prescribing decision; and (iii) culture-guided (definitive) therapy, when antibiotics were initiated or substantially modified after a pathogen had been identified and/or susceptibility data were available. Only scenario (iii) was classified as “culture-guided” in descriptive analyses.

#### 4.4.2. Secondary Infections

Secondary infections included bloodstream infection, ventilator-associated pneumonia (VAP), hospital-acquired pneumonia (HAP), catheter-associated urinary tract infection, and *Clostridioides difficile* infection. Each episode required both compatible clinical criteria and microbiological confirmation and was classified as hospital-acquired if identified ≥48 h after admission, consistent with CDC and ECDC surveillance criteria. For VAP and HAP, we applied definitions aligned with ATS/IDSA and CDC/NHSN frameworks, requiring a new or progressive radiological infiltrate plus at least two of the following: fever or hypothermia, leukocytosis or leukopenia, purulent respiratory secretions, or worsening oxygenation, in conjunction with a positive quantitative or semi-quantitative culture from lower respiratory tract specimens (endotracheal aspirate or bronchoalveolar lavage) judged by the treating team to represent true infection rather than colonization. Catheter-associated urinary tract infection required urinary symptoms or systemic signs compatible with infection, pyuria, and a urine culture growing ≥10^5^ CFU/mL of a uropathogen from a patient with an indwelling urinary catheter. Bloodstream infection was defined as at least one positive blood culture for a pathogen (with standard criteria used to distinguish true infection from common skin contaminant isolates), in combination with clinical signs of sepsis. *Clostridioides difficile* infection required diarrhea plus a positive toxin assay and/or toxigenic C. difficile detected by PCR. Multidrug-resistant organisms (MDROs) were defined according to international consensus as bacterial isolates non-susceptible to at least one agent in three or more antimicrobial classes.

Infections were classified as secondary if they occurred during the index hospitalization in patients without documented active infection at admission. Because the exact onset date of secondary infections (clinical or microbiological) was not systematically recorded, we could not reliably determine the time elapsed between antibiotic initiation and infection onset. Therefore, temporal analyses focus on the presence of secondary infection during hospitalization rather than on precise timing. In most cases, antibiotics were initiated in response to respiratory deterioration or elevated inflammatory markers suggestive of COVID-19 progression rather than proven bacterial infection; however, some degree of reverse causation cannot be excluded.

#### 4.4.3. Severity of COVID-19

Disease severity was categorized according to NIH and WHO criteria, incorporating the need for oxygen supplementation, radiological findings, and organ dysfunction parameters. NEWS2 and SOFA scores were not systematically computed for the cohort for the reasons detailed in the Data Sources and Variables Collected subsection.

### 4.5. Microbiological Procedures

Blood cultures were processed using automated continuous-monitoring systems. Respiratory specimens (sputum, endotracheal aspirates, bronchoalveolar lavage) and urine samples were cultured on standard media and incubated according to EUCAST recommendations. For respiratory specimens, culture results were interpreted in conjunction with clinical and radiological findings. Growth from surveillance swabs or respiratory cultures obtained in clinically stable patients without new or progressive infiltrates or systemic signs of infection was considered colonization and was not classified as a secondary infection. Only episodes fulfilling the clinical, radiological, and microbiological criteria summarized above were counted as respiratory secondary infections in the primary analyses. Identification and antimicrobial susceptibility testing were performed using MALDI-TOF MS and automated microdilution systems. All susceptibility results were interpreted using contemporary EUCAST breakpoints for the study year.

Microbiological sampling followed routine clinical practice in our tertiary infectious diseases center and was not protocolized by the study team. Blood cultures, respiratory samples and urine cultures were obtained at the discretion of treating physicians, typically in patients with clinical deterioration, new or persistent fever, hemodynamic instability, rising inflammatory markers, or new radiological infiltrates. Although our hospital has a low threshold for obtaining cultures in moderate-to-severe COVID-19, more severely ill patients, those admitted to the ICU, and those receiving invasive mechanical ventilation were more likely to undergo repeated and invasive sampling, which may increase the probability of detecting secondary infections and multidrug-resistant organisms. Because culture ordering was clinician-driven and more intensive in sicker patients, the primary outcome of microbiologically confirmed secondary infection is inherently susceptible to detection bias.

*Clostridioides difficile* infection was diagnosed using a two-step algorithm incorporating GDH antigen and toxin A/B enzyme immunoassay, with PCR confirmation in discordant cases.

### 4.6. Outcomes

The primary outcome was the occurrence of a microbiologically confirmed secondary infection during hospitalization.

Secondary outcomes included ICU admission, the need for invasive mechanical ventilation, length of stay, and in-hospital mortality. In addition, we evaluated a composite MDRO colonization or infection endpoint, defined as the isolation of an MDRO from any clinical or surveillance culture during hospitalization, irrespective of whether the isolate fulfilled criteria for a secondary infection episode. We additionally summarized the distribution of microbiologically confirmed bloodstream infection across ICU admission and antibiotic exposure strata as a descriptive supplementary analysis (Supplementary Materials, Table S1). Given the retrospective nature of the dataset, detailed information on the exact number and timing of cultures per patient was not systematically available and could not be robustly quantified.

### 4.7. Statistical Analysis

Continuous variables were summarized as mean ± standard deviation or median (interquartile range), depending on distribution. Categorical variables were described as frequencies and percentages. Between-group comparisons were performed using Student’s *t*-test or Mann–Whitney U-test for continuous variables and χ^2^ or Fisher’s exact test for categorical variables.

Multivariable logistic regression models were used to evaluate the association between antibiotic exposure and secondary infections, adjusting for age, comorbidities, baseline COVID-19 severity (NIH/WHO category, oxygen requirement, and admission inflammatory biomarkers), corticosteroid use, and duration of hospitalization. We included length of stay in these models to partially account for differences in time at risk for developing hospital-acquired infections, acknowledging that duration of hospitalization may also lie on the causal pathway between antibiotic exposure, disease severity, and downstream outcomes. Additional models examined the relationship between antibiotic patterns (class, timing, duration) and major clinical outcomes. Model assumptions were verified, and results were expressed as adjusted odds ratios (aORs) with 95% confidence intervals. Multicollinearity among covariates was assessed using variance inflation factors (VIF), with all values <2 indicating low collinearity. Proportional hazards assumptions in Cox models were verified using Schoenfeld residuals. Analyses were performed using a complete-case approach. Key covariates included in multivariable models (age, comorbidities, NIH/WHO COVID-19 severity category, oxygen requirement, and admission inflammatory biomarkers) had low levels of missingness; therefore, no formal imputation procedures were applied. Patients with missing values in any of these variables were excluded from the corresponding adjusted analyses, and sample sizes are reported in the tables for each model.

To further address potential selection bias related to microbiological data availability, we conducted sensitivity analyses: (a) restricting the cohort to patients with hospitalization ≥5 days (reducing the likelihood of missing hospital-acquired events in short-stay cases); and (b) excluding patients without documented blood cultures as a stricter proxy for diagnostic workup. To further assess the impact of differential diagnostic intensity, we performed a sensitivity analysis restricted to patients who underwent microbiological sampling, operationalized as having at least one documented blood culture during hospitalization. In this restricted cohort, the multivariable association between any antibiotic exposure and secondary infection remained materially unchanged, with adjusted odds ratios that were highly similar to the main analysis. Results remained consistent with the primary analyses, with similar adjusted odds ratios for the association between antibiotic exposure and secondary infection in both sensitivity models.

To further address potential selection bias related to microbiological data availability, we conducted sensitivity analyses: (a) restricting the cohort to patients with hospitalization ≥5 days (reducing the likelihood of missing hospital-acquired events in short-stay cases); and (b) restricting the cohort to patients with documented microbiological sampling, operationalized as having at least one blood culture during hospitalization. In this restricted cohort, the multivariable association between any antibiotic exposure and secondary infection remained materially unchanged, with adjusted odds ratios that were highly similar to the main analysis. In addition, as a descriptive supplementary analysis, we summarized the distribution of microbiologically confirmed bloodstream infection across ICU admission and antibiotic-exposure strata (Supplementary Materials, Table S1). Results remained consistent with the primary analyses, with similar adjusted odds ratios for the association between antibiotic exposure and secondary infection in both sensitivity models.

Survival analyses (Kaplan–Meier curves and Cox proportional hazards models) were performed for time-to-event outcomes when appropriate. Statistical significance was defined as *p* < 0.05. Analyses were performed using SPSS v.26 (IBM Corp., Armonk, NY, USA) and R v.4.3.0 (R Foundation for Statistical Computing, Vienna, Austria). For time-to-event analyses, hospitalization duration was used as the time scale because the exact onset date of secondary infections was not systematically recorded; events were therefore defined using infection-related endpoints available in the dataset (sepsis and/or a documented bloodstream isolate).

### 4.8. Ethical Considerations

The study was conducted in accordance with the Declaration of Helsinki and was approved by the Ethics Committee of Victor Babeș University of Medicine and Pharmacy and the Victor Babeș Clinical Hospital for Infectious Diseases (approval no. 70/1 September 2022; revised approval no. 2174/10 March 2023). In our institution, a standardized admission form includes a broad consent clause whereby patients (or, when applicable, their legally authorized representatives) authorize the secondary use of de-identified clinical data for research and quality-improvement purposes in accordance with institutional policy. For this retrospective analysis, no additional study-specific informed consent was sought, as all patients (or their legally authorized representatives) had already provided written informed consent for the secondary use of anonymized clinical data at hospital admission, and no identifiable information was collected or reported.

## 5. Conclusions

In this real-world cohort of hospitalized adults with COVID-19, antibiotic exposure was widespread, predominantly empirical, and only partially aligned with the relatively modest burden of microbiologically confirmed bacterial co-infection at admission. At the same time, secondary infections were largely hospital-acquired and frequently caused by multidrug-resistant organisms.

Prolonged antibiotic therapy, early initiation, and preferential use of WHO Watch/Reserve agents were independently associated with higher odds of secondary infection, MDRO colonization or infection, and adverse clinical outcomes, including ICU admission, invasive mechanical ventilation, longer hospitalization, and in-hospital mortality.

These data support a risk-amplification paradigm in which broad and extended antibiotic exposure during severe viral respiratory illness may contribute to downstream nosocomial superinfection and resistance selection, rather than functioning solely as a marker of baseline disease severity.

From a clinical perspective, stewardship strategies in COVID-19 and future viral respiratory epidemics should give priority to restrictive use of early broad-spectrum therapy, systematic 48–72 h reassessment with de-escalation or discontinuation when bacterial infection is not supported, preferential use of Access agents when appropriate, and strict limitation of treatment duration beyond seven days.

Integrating these antibiotic-optimization measures with strong microbiological diagnostics and infection prevention and control programs is essential to reduce avoidable infectious complications, curb the hospital MDRO burden, and preserve the effectiveness of existing antibacterials in resource-limited acute-care settings.

## Figures and Tables

**Table 1:** Baseline demographic, clinical and laboratory characteristics of the study population on admission (*n* = 395). Standardized NEWS2 and SOFA scores were not systematically available because several key components were missing for a significant proportion of patients; severity on admission is therefore summarized according to NIH/WHO COVID-19 categories, oxygen requirement, SpO_2_ and biomarker profiles.

Characteristic	Value
Demographics	
Age, years, mean ± SD	70.3 ± 11.2
Male sex, *n* (%)	230 (58.2)
Urban residence, *n* (%)	241 (61.0)
Body mass index (BMI), kg/m^2^, mean ± SD	29.4 ± 5.6
Obesity (BMI ≥ 30 kg/m^2^), *n* (%)	168 (42.5)
Vaccination status	
Fully vaccinated (≥2 doses), *n* (%)	190 (48.1)
Partially vaccinated (1 dose), *n* (%)	49 (12.4)
Unvaccinated, *n* (%)	156 (39.5)
Smoking and immunosuppression	
Ever smoker, *n* (%)	96 (24.3)
Immunosuppressed, *n* (%)	36 (9.1)
Comorbidities	
Cardiovascular disease, *n* (%)	246 (62.3)
Arterial hypertension, *n* (%)	232 (58.7)
Ischemic heart disease, *n* (%)	111 (28.1)
Diabetes mellitus, *n* (%)	129 (32.7)
Type 2 diabetes, *n* (%)	116 (89.9) ᵃ
Type 1 diabetes, *n* (%)	10 (7.8) ᵃ
Newly diagnosed at admission, *n* (%)	3 (2.3) ᵃ
Chronic kidney disease, *n* (%)	71 (18.0)
Chronic obstructive pulmonary disease, *n* (%)	56 (14.2)
COVID-19 severity at admission	
Moderate-to-severe disease ᵇ, *n* (%)	270 (68.4)
SpO_2_ on admission, %, mean ± SD	90.2 ± 3.8
Oxygen supplementation required at admission, *n* (%)	366 (92.7)
Laboratory markers at admission (mean ± SD)	
Leukocyte count, ×10^3^/µL	9.8 ± 4.7
C-reactive protein (CRP), mg/L	98.5 ± 72.3
Procalcitonin, ng/mL	0.52 ± 0.81
Ferritin, ng/mL	812.4 ± 592.6
D-dimer, µg/mL	1.42 ± 1.18
Interleukin-6 (IL-6), pg/mL	42.3 ± 38.7
Elevated inflammatory marker ^c^, *n* (%)	334 (84.6)

Note: ᵃ Percentage calculated within the diabetes subgroup (*n* = 129). ᵇ According to NIH/WHO severity criteria. ^c^ At least one of: elevated CRP, procalcitonin, ferritin, IL-6, or D-dimer above local reference range. Data are present-ed as *n* (%), mean ± standard deviation, or as otherwise indicated.

**Table 2:** Antibiotic prescribing patterns in the study cohort.

Characteristic	Value
Patients receiving systemic antibiotics, *n* (%)	349 (88.4)
Timing of antibiotic initiation	
Early initiation (≤24 h from admission), *n* (%)	253 (72.5)
Late initiation (>24 h from admission), *n* (%)	96 (27.5)
Treatment duration	
Mean ± SD, days	8.2 ± 3.1
Median (IQR), days	7 (5–10)
Regimen structure	
Monotherapy, *n* (%)	144 (41.3)
Combination therapy, *n* (%)	205 (58.7)
Treatment modifications	
Escalation occurred, *n* (%)	112 (32.1)
De-escalation occurred, *n* (%)	75 (21.5)
WHO AWaRe classification (among exposed patients)	
Access antibiotics used, *n* (%)	99 (28.4)
Watch antibiotics used, *n* (%)	191 (54.7)
Reserve antibiotics used, *n* (%)	59 (16.9)
Most commonly prescribed agents	
Ceftriaxone, *n* (%)	133 (38.1)
Moxifloxacin, *n* (%)	86 (24.6)
Meropenem, *n* (%)	64 (18.3)
Piperacillin–tazobactam, *n* (%)	42 (12.0)
Type of prescribing	
Empirical (initiated without microbiological confirmation), *n* (%)	284 (81.4)
Culture-guided, *n* (%)	65 (18.6)

Note: Percentages for AWaRe classes, most common agents, and type of prescribing are calculated among the 349 patients who received systemic antibiotics. Data are presented as *n* (%), mean ± standard deviation, or median (interquartile range) as appropriate. Empirical therapy was defined as antibiotic initiation without microbiological or susceptibility results available at the time of the prescribing decision (including fully empirical starts and empirical therapy with cultures pending), whereas culture-guided therapy referred to initiation or major modification after pathogen identification and/or susceptibility data were available.

**Table 3:** Characteristics of microbiologically confirmed secondary infections (*n* = 112).

Characteristic	Value
Patients with secondary infection, *n* (%)	112 (28.4)
Timing/Acquisition	
Hospital-acquired (>48 h after admission), *n* (%)	92 (82.1)
Clinical syndromes	
Ventilator-associated pneumonia, *n* (%)	39 (34.8)
Hospital-acquired pneumonia, *n* (%)	33 (29.5)
Bloodstream infection, *n* (%)	21 (18.8)
Urinary tract infection, *n* (%)	14 (12.5)
*Clostridioides difficile* infection, *n* (%)	5 (4.5)
Multidrug-resistant organisms (MDRO)	
MDRO isolated in secondary infections, *n* (%)	46 (41.1)
Most common MDRO pathogens (among 46 MDRO cases)	
ESBL/KPC/OXA-48–producing *Klebsiella pneumoniae*, *n* (%)	20 (43.5)
Multidrug-resistant *Pseudomonas aeruginosa*, *n* (%)	12 (26.1)
Vancomycin-resistant *Enterococcus faecium*, *n* (%)	7 (15.2)
Carbapenem-resistant *Acinetobacter baumannii*, *n* (%)	6 (13.0)

Note: Percentages for timing, clinical syndromes, and MDRO are calculated among the 112 patients with microbiologically confirmed secondary infections. MDRO pathogen percentages are calculated among the 46 cases involving multidrug-resistant organisms. Data are presented as *n* (%).

**Table 4:** Clinical outcomes of the study population (*n* = 395).

Outcome/Complication	Value
Primary and major secondary outcomes	
Microbiologically confirmed secondary infection, *n* (%)	112 (28.4)
ICU admission, *n* (%)	87 (22.0)
ICU length of stay, days, mean ± SD	8.9 ± 5.3
Invasive mechanical ventilation, *n* (%)	62 (15.7)
Duration of invasive mechanical ventilation, days, mean ± SD	7.2 ± 4.1
In-hospital length of stay, days, mean ± SD	10.4 ± 6.8
In-hospital length of stay, days, median (IQR)	9 (5–14)
In-hospital mortality, *n* (%)	50 (12.7)
Other complications	
Pulmonary complications (pleural effusion, thromboembolic events), *n* (%)	151 (38.2)
Sepsis or septic shock, *n* (%)	59 (14.9)
Urinary tract infection (not meeting secondary infection criteria), *n* (%)	36 (9.1)
Enterocolitis, *n* (%)	21 (5.3)
In-hospital cardiac arrest, *n* (%)	16 (4.1)

Notes: Data are presented as *n* (%), mean ± standard deviation, or median (interquartile range) as appropriate.

**Table 5:** Multivariable associations between antibiotic exposure patterns and key clinical outcomes.

Outcome/Exposure Variable	Adjusted OR (95% CI)	*p*-Value
Primary outcome: Microbiologically confirmed secondary infection		
Any antibiotic exposure	2.15 (1.42–3.27)	<0.001
Antibiotic duration ≥7 days	1.82 (1.21–2.74)	0.004
Use of Watch/Reserve antibiotics	1.97 (1.35–2.88)	<0.001
Early initiation (≤24 h from admission)	1.48 (1.02–2.15)	0.039
Exposure to Reserve antibiotics (MDRO risk)	2.31 (1.56–3.42)	<0.001
Secondary clinical outcomes		
ICU admission	1.76 (1.18–2.62)	0.005
Invasive mechanical ventilation	1.93 (1.27–2.94)	0.002
Prolonged hospital stay (>10 days)	1.64 (1.12–2.41)	0.011
In-hospital mortality	1.89 (1.22–2.93)	0.004

## Data Availability

De-identified clinical, laboratory, and imaging data supporting the findings of this study are available from the corresponding authors upon reasonable request, subject to institutional data-sharing policies.

## Supplementary Materials

The following supporting information can be downloaded at: https://www.mdpi.com/article/10.3390/antibiotics15030240/s1, Figure S1: STROBE-style flow diagram of cohort assembly; Table S1: Frequency of bloodstream infection across key clinical strata (ICU admission and antibiotic exposure); Table S2: Admission inflammatory biomarkers according to antibiotic exposure.

## Abbreviations

The following abbreviations are used in this manuscript:

AMR	Antimicrobial resistance
aOR	Adjusted odds ratio
AWaRe	Access, Watch, and Reserve (WHO antibiotic classification)
BAL	Bronchoalveolar lavage
BMI	Body mass index
CDC	Centers for Disease Control and Prevention
CI	Confidence interval
CKD	Chronic kidney disease
COVID-19	Coronavirus disease 2019
CRP	C-reactive protein
DOT	Days of therapy
ECDC	European Centre for Disease Prevention and Control
ESBL	Extended-spectrum beta-lactamase
EUCAST	European Committee on Antimicrobial Susceptibility Testing
GDH	Glutamate dehydrogenase
HAI	Hospital-acquired infection
HR	Hazard ratio
ICU	Intensive care unit
IL-6	Interleukin-6
IQR	Interquartile range
MDRO	Multidrug-resistant organism
MDR	Multidrug resistant
NIH	National Institutes of Health
NEWS2	National Early Warning Score 2
PCR	Polymerase chain reaction
RT-PCR	Reverse transcription polymerase chain reaction
SD	Standard deviation
SOFA	Sequential Organ Failure Assessment
SpO_2_	Peripheral oxygen saturation
UTI	Urinary tract infection
VAP	Ventilator-associated pneumonia
WHO	World Health Organization
