# Supplementary material for: Patterns of Antibiotic Use in Hospitalized COVID-19 Patients and Association with Secondary Infections and Outcomes
Source: Antibiotics (Basel). 2026 Feb 25;15(3):240. doi: 10.3390/antibiotics15030240 (PMC13024623; doi:10.3390/antibiotics15030240)
Supplement: Supplementary file 1 [file antibiotics-15-00240-s001.zip › antibiotics-4165077-supplementary.pdf]

Supplementary Materials

Supplementary Figure S1. STROBE-style flow diagram of cohort assembly.

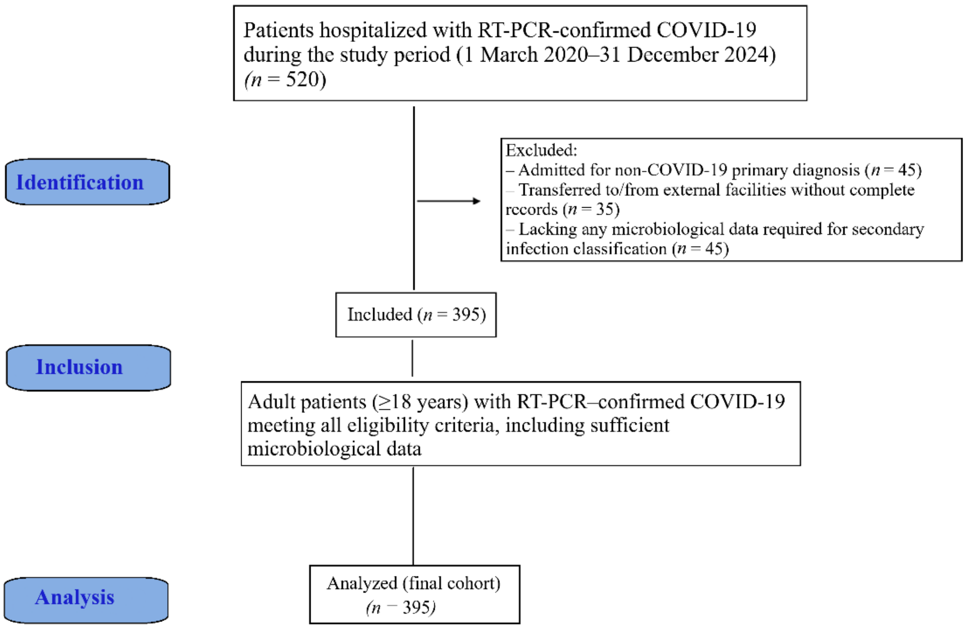

Supplementary Figure S1. STROBE-style flow diagram of cohort assembly. The figure summarizes screening of 520 adults hospitalized with RT-PCR–confirmed COVID-19 during the study period, application of inclusion and exclusion criteria (non-COVID-19 primary diagnosis, transfer without complete records, and lack of microbiological data required for secondary infection classification), and inclusion of the final analytic cohort of 395 patients who met all eligibility criteria and had sufficient microbiological data.

Supplementary Table S1. Frequency of bloodstream infection across key clinical strata (ICU admission and antibiotic exposure).

Group	N	Patients with microbiologically confirmed bloodstream infection, n (%)
ICU admission – Yes	21	0 (0.0)
ICU admission – No	374	27 (7.2)
Antibiotic exposure – Yes	285	20 (7.0)
Antibiotic exposure – No	110	7 (6.4)

Supplementary Table S2. Admission inflammatory biomarkers according to antibiotic exposure.

Variable	Antibiotic exposure (n = 285)	No antibiotic exposure (n = 110)	p-value
C-reactive protein (CRP), mg/L	181.39 ± 942.77	43.50 ± 37.05	<0.001
Procalcitonin, ng/mL	1.25 ± 2.52	0.30 ± 0.32	<0.001
Ferritin, ng/mL	882.84 ± 675.60	329.75 ± 271.54	<0.001
D-dimer, μg/mL	2.07 ± 11.26	0.77 ± 0.40	0.079
Interleukin-6 (IL-6), pg/mL	39.84 ± 75.65	7.17 ± 7.48	<0.001
Leukocyte count, $\times 10^3/\mu L$	14.06 ± 4.65	7.37 ± 2.57	<0.001
